# miR-34a-5p as molecular hub of pathomechanisms in Huntington’s disease

**DOI:** 10.1186/s10020-023-00640-7

**Published:** 2023-04-03

**Authors:** Martin Hart, Caroline Diener, Laetitia Lunkes, Stefanie Rheinheimer, Lena Krammes, Andreas Keller, Eckart Meese

**Affiliations:** 1 Institute of Human Genetics, Saarland University, Building 60, 66421 Homburg, Germany; 2 Chair for Clinical Bioinformatics, Saarland University, 66123 Saarbrücken, Germany; 3 Department of Neurology and Neurological Sciences, Stanford University School of Medicine, Stanford, CA USA

**Keywords:** miR-34a-5p, Huntington’s disease, NDUFA9, HIP1, TGM2, POLR2G, HD pathomechanisms, miR-based therapy

## Abstract

**Background:**

Although a pivotal role of microRNA (miRNA, miR) in the pathogenesis of Huntington’s disease (HD) is increasingly recognized, the molecular functions of miRNAs in the pathomechanisms of HD await further elucidation. One of the miRNAs that have been associated with HD is miR-34a-5p, which was deregulated in the mouse R6/2 model and in human HD brain tissues.

**Methods:**

The aim of our study was to demonstrate interactions between miR-34a-5p and HD associated genes. By computational means we predicted 12 801 potential target genes of miR-34a-5p. An in-silico pathway analysis revealed 22 potential miR-34a-5p target genes in the KEGG (Kyoto Encyclopedia of Genes and Genomes) pathway “Huntington’s disease”.

**Results:**

Using our high-throughput miRNA interaction reporter assay (HiTmIR) we identified *NDUFA9, TAF4B, NRF1, POLR2J2, DNALI1, HIP1, TGM2* and *POLR2G* as direct miR-34a-5p target genes. Direct binding of miR-34a-5p to target sites in the 3’UTRs of *TAF4B, NDUFA9, HIP1* and *NRF1* was verified by a mutagenesis HiTmIR assay and by determining endogenous protein levels for HIP1 and NDUFA9*.* STRING (Search Tool for the Retrieval of Interacting Genes/Proteins) analysis identified protein–protein interaction networks associated with HD like “Glutamine Receptor Signaling Pathway” and “Calcium Ion Transmembrane Import Into Cytosol”.

**Conclusion:**

Our study demonstrates multiple interactions between miR-34a-5p and HD associated target genes and thereby lays the ground for future therapeutic interventions using this miRNA.

**Supplementary Information:**

The online version contains supplementary material available at 10.1186/s10020-023-00640-7.

## Background

Huntington’s disease (HD) is a rare, dominantly-inherited, neurodegenerative disorder and is caused by an abnormal amplification of CAG repeats (encoding for glutamine) in the huntingtin (HTT) gene (The Huntington’s Disease Collaborative Research Group 1993; MacDonald et al. 1993). These polyglutamine repeats lead to misfolding and aggregation of the mutant Huntingtin (mHTT) causing neurotoxicity (Cattaneo et al. 2005). MiRNAs belong to a class of small non-coding RNAs, are ~ 22 nucleotides (nt) in length (Ambros et al. 2003), and regulate the expression of their target mRNAs post-transcriptionally by binding with their seed-sequence to complementary binding sites in their 3’UTRs (Engels and Hutvagner 2006). In very rare cases miRNAs can bind to sequences of 5’ untranslated regions or open reading frames of their target genes (Moretti et al. 2010).

The molecular functions of miRNAs in the pathogenesis of HD are only partially understood. Until now, there is a lack of a comprehensive analysis of miRNA function in the regulation of Huntington pathways in general. Since the first description of the association between HD pathogenesis and miRNA dysregulation in 2008 (Johnson et al. 2008) relatively few publications analyzed miRNA-target gene networks. Out of 14,252 total publications dealing with HD, only 249 (1.7%) analyzed the role of miRNAs in the pathogenesis of HD compared to other diseases like for example lung cancer where 8123 of 143,495 total publications (5.7%) dealt with miRNAs. However, the pivotal roles of miRNAs in the pathogenesis of a variety of different neurological disorders such as Parkinson’s, Alzheimer’s and Huntington’s disease are well recognized (Cardo et al. 2013; Leidinger et al. 2013; Reynolds et al. 2018).

Reynolds et al. found decreased levels of miR-34a-5p in brain tissue of R6/2 mouse model of HD. This study identified a positive feedback loop of p53, miR-34a-family and SIRT1 with impact on cell cycle progression, cellular senescence and apoptosis (Reynolds et al. 2018). Additionally, the re-analysis of next generation small RNA sequencing data of Hoss et al. (Hoss et al. 2015) showed a significant down-regulation of miR-34a-5p in human HD brain samples. We recently determined the strong impact of miR-34a-5p on the regulation of crucial Parkinson’s disease associated pathways, like “dopaminergic synapse”, “dopamine receptor signaling” or “pink/parkin mediated mitophagy” (Kern et al. 2021). So, there is ample evidence that miR-34a-5p can target complex regulation networks in neurodegeneration. Using our previously developed method called high-throughput miRNA interaction reporter assay (HiTmIR), we set out for a comprehensive analysis of miR-34a-5p function in HD to unravel underlying key pathomechanisms. We used this combined computational and experimental approach to identify miR-34a-5p target genes in Huntington’s disease pathways.

## Methods

### Cell lines

The human cell lines HEK 293T (ACC 635) and SH-SY5Y (ACC 209) were purchased from the German collection of microorganisms and cell cultures (DSMZ). Both cell lines were authenticated by STR typing by the supplier. The HEK 293T cells were cultured in DMEM (Life Technologies, Darmstadt, Germany) with Penicillin (100U/ml), Streptomycin (100 µg/ml) and 10% [v/v] FCS and sub-cultured two times a week. The SH-SY5Y cells were cultured in DMEM (Life Technologies, Darmstadt, Germany) with Penicillin (100 U/ml), Streptomycin (100 µg/ml) and 20% [v/v] FCS and sub-cultured two times a week. Both cell lines were cultured for less than 3 months after receipt.

### miRNA expression plasmid and reporter vectors

The pSG5-mir-34a expression vector was synthesized and cloned by Eurofins Genomics and includes the nucleotides 9,151,617–9,151,816 of chromosome 1 (Eurofins Genomics, Ebersberg, Germany). The expression of miR-34a-5p from this expression construct was verified by Northern Blotting and described previously (Hart et al. 2016). The 3’UTR sequences of *NDUFA9_2*, *TAF4B*, *NRF1*, *POLR2J2_1*, *NDUFA9_1*, *DNALI1*, *HIP1*, *TGM2*, *POLR2G*, *NDUFA4L2*, *POLR2H*, *SP1_2*, *POLR2E*, *SP1_1*, *IFT57*, *PPARG*, *HAP1*, *SOD2*, *POLR2J3*, *CREB3L3*, *POLR2J2_2*, *POLR2F*, *RCOR1*, *DNAL4* and *UQCRFS1* were PCR amplified with specific primers and cloned via *Spe*I, *Sac*I or *Nae*I restriction sites into the pMIR-RNL-TK vector, which was described in Beitzinger et al. (Beitzinger et al. 2007). For target genes with long 3’UTRs (> 1 500 nt) and more than one predicted miR-34a-5p binding site the 3’UTR sequences were split into two fragments. This was the case for NDUFA9, SP1, POLR2J2. The identifiers of all cloned 3’UTR (NM accession numbers) and the sequences of the respective cloning primers are given in Additional file 4: table S1. For validation of the positively tested target genes respective binding sites were mutated using specific mutagenesis primers via overlap extension PCR. The sequences of the mutagenesis primers are given in Additional file 5: table S2.

### High-throughput miRNA interaction reporter assay (HiTmIR)

The *in-silico* prediction of miR-34a-5p target genes were conducted with miRWalk 2.0 and the *in-silico* pathway analysis of the predicted miR-34a-5p target genes were performed with GeneTrail3 (Dweep and Gretz 2015; Gerstner et al. 2020). The HiTmIR-Assay was described by our group previously (Kern et al. 2021). In brief, 3.2 × 10^4^ HEK 293T cells were seeded out per well of a 96-well plate using the liquid handling system epMotion^®^ 5075 (Eppendorf, Hamburg, Germany). Next day the cells were transfected with 50 ng/well of either reporter plasmid pMIR-RNL-TK, with or without insert, and 200 ng/well of miRNA expression plasmid containing either the respective miRNA or no insert. 48 h later the transfected cells were lysed and measured using the Dual-Luciferase^®^ Reporter Assay System (Promega, Madison, WI, USA) on a GloMax Navigator microplate luminometer (Promega, Madison, WI, USA). For analysis the firefly luciferase activity of each wild type 3’UTR reporter construct was standardized using a constitutively expressed renilla luciferase (Ratio Firefly/Renilla). The standardized luciferase activity of each wild type 3’UTR reporter construct co-transfected with miR-34a was normalized to the luciferase activity of the empty reporter vector co-transfected with miR-34a. The HiTmIR-Assay were conducted four times in technical duplicates. The statistical analysis of the reporter assays was performed with GraphPad Prism 9 applying the Welsh’s t-test. The pMIR-TCRA reporter plasmid served as positive control and was described previously (Hart et al. 2018).

### Mutagenesis high-throughput miRNA interaction reporter assay (Mutagenesis HiTmIR)

The Mutagenesis HiTmIR-Assay was described above. In brief, 3.2 × 10^4^ HEK 293T cells were seeded out per well of a 96-well plate using the liquid handling system epMotion^®^ 5075 (Eppendorf, Hamburg, Germany). Next day the cells were transfected with 50 ng/well of either reporter plasmid pMIR-RNL-TK, with or without or mutated insert and 200 ng/well of miRNA expression plasmid containing either the respective miRNA or no insert. 48 h later the transfected cells were lysed and measured using the Dual-Luciferase^®^ Reporter Assay System (Promega, Madison, WI, USA) on a GloMax Navigator microplate luminometer (Promega, Madison, WI, USA). The Mutagenesis HiTmIR-Assays were conducted in three independent experiments with technical duplicates. The statistical analysis of the reporter assays was performed with GraphPad Prism 9 applying the Welsh’s t-test. The pMIR-TCRA reporter plasmid served as positive control and was described previously (Hart et al. 2018).

### Western blot

For analysis of the protein level of targets genes upon miR-34a-5p over-expression, 4.5 × 10^5^ SH-SY5Y cells per well of a 6-well plate were seeded out. Next day, cells were transfected with either Allstars Negative Control (ANC; Qiagen, Hilden, Germany) or hsa-miR-34-5p miScript miRNA Mimic (MIMAT0000255: 5′UGGCAGUGUCUUAGCUGGUUGU3′; Qiagen, Hilden, Germany) using HiPerFect Transfection Reagent (Qiagen, Hilden, Germany). 48 h post transfection cells were harvested and lysed in 2 × sample buffer (130 mM Tris/HCl, 6% [v/v] SDS, 10% [v/v] 3-Mercapto-1,2-propandiol, 10% [v/v] glycerol) and 3 times sonicated for 3 s. 15 µg of the whole protein extracts were separated using a 4–15% TGX gel (Bio-Rad Laboratories Inc., Hercules, California, USA). The separated proteins were electroblotted onto nitrocellulose membrane (Whatman, GE Healthcare, Freiburg, Germany). After Blotting the membrane was blocked by pre-incubation with 5% TBS milk with 0.1% Tween 20 for 30 min to avoid unspecific antibody binding. HIP1 was detected by monoclonal mouse antibody (13E1, Thermo Fisher, Waltham, USA) and NDUFA9 by monoclonal mouse antibody (20C11B11B11, Thermo Fisher, Waltham, USA). α-Tubulin served as endogenous controls and was detected by monoclonal rabbit antibody (#2125, Cell Signaling Technology, Danvers, USA). The secondary anti-rabbit and anti-mouse antibodies were purchased from Sigma-Aldrich (A0545, A9044, Sigma Aldrich, Munich, Germany).

### Data analysis and web tools

Statistical analysis of the luciferase assays, the Western blots were conducted with GraphPad Prism 9 (GraphPad Software, La Jolla, USA) applying the Welsh’s t-test. Quantification of the Western blots was performed with Image Lab Software Version 6.0.1 (Bio-Rad Laboratories Inc., Hercules, USA). The asterisks in the figures correspond to the statistical significance as calculated by Welsh’s t-test: * = 0.01 < p ≤ 0.05; ** = 0.001 < p ≤ 0.01; *** = p ≤ 0.001. GeneTrail3 was used for the pathway analysis of predicted target genes by an over-representation analysis (ORA) with default settings (Null hypothesis (for p-value computation): Two-sided; Method to adjust p-values: Benjamini-Yekutieli; Significance level: 0.05; reference set: all protein coding genes) (http://genetrail.bioinf.uni-sb.de/) (Gerstner et al. 2020). For the protein–protein interaction network analysis we used the STRING database version 11.5 (https://string-db.org/) with default settings for all validated miR-34a-5p target genes in the KEGG “Huntington’s Disease pathway” (Szklarczyk et al. 2019). miRWalk 2.0 was used for in-silico target prediction of miR-34a-5p target genes. This tool comprised 10 algorithms including DIANAmT3.0, miRanda (2010), miRDB (2009), miRWalk, RNAhybrid (version2.1), PICTAR4 (2006), PICTAR5 (2007), PITA (2008), RNA22 (2008), and TargetScan5.1 (Dweep and Gretz 2015).

## Results

### Prediction of miR-34a-5p target genes associated with Huntington’s disease

**Fig. 1:**
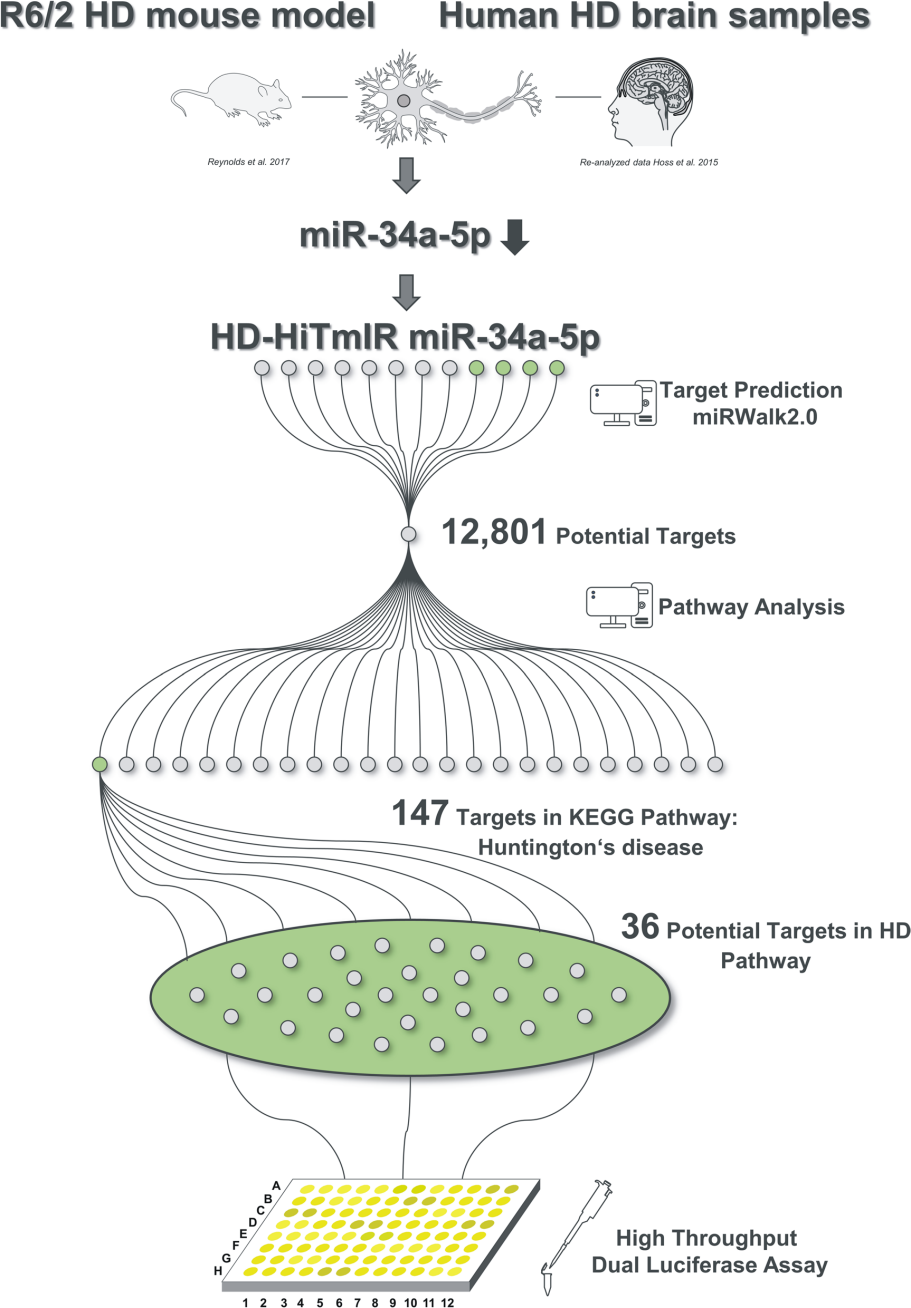
Workflow for comprehensive identification of miR-34a-5p target networks in Huntington’s disease. Reynolds et al. described a decreased miR-34a-5p expression in R6/2 HD mouse model (Reynolds et al. 2018). An *in-silico* target prediction by miRwalk2.0 identified 12 801 potential miR-34a-5p target genes. An overrepresentation analysis of these potential target genes assigned 147 potential target genes to the KEGG pathway “Huntington’s Disease”. Out of these target genes we excluded already validated target genes of miRTarBase 8.0 and target genes without canonical miR-34a-5p binding sites. Out of the remaining 36 target genes, 14 target genes were already tested in Kern et al. (Kern et al. 2021) and 22 target genes were tested in this study using our automated dual luciferase reporter assay

In 2018 Reynolds et al. found miR-34a-5p down-regulated in brain tissue of R6/2 mouse model of HD (Reynolds et al. 2018). Additionally, we re-analyzed next generation small RNA sequencing data of Hoss et al. including 28 Huntington’s Disease brain samples and 36 neurologically normal human post-mortem prefrontal cortex (BA9) brain samples (GSE64977) (Hoss et al. 2015) for miR-34a-5p expression. Thereby we detected a significant downregulation of miR-34a-5p in human HD brain samples (Additional file 1: Fig. S1). To comprehensively investigate the overall importance of miR-34a-5p in HD we conducted an in silico prediction of target genes of miR-34a-5p using miRWalk 2.0 (Dweep and Gretz 2015), which comprised 10 algorithms including DIANAmT, miRanda, miRDB, miRWalk, RNAhybrid, PICTAR4, PICTAR5, PITA, RNA22 and Targetscan. This prediction identified 12 801 potential miR-34a-5p target genes. Using GeneTrail3 (https://genetrail2.bioinf.uni-sb.de/), a web service allowing the integrated analysis of transcriptomic, miRNomic, genomic and proteomic datasets, we identified 147 miR-34a-5p target genes which were significantly enriched in the KEGG pathway “Huntington’s Disease” (p ≤ 0.05) Additional files 6, 7: table S3 and table S4   (Gerstner et al. 2020). We refined this list to a number of 36 miR-34a-5p target genes by depletion of target genes, which were already published according to miRTarBase 8.0 (Huang et al. 2020) and of target genes without canonical miR-34a-5p binding sites. Out of the remaining 36 target genes, we already tested 14 PD-associated miR-34a-5p target genes in our previous study (Kern et al. 2021). The remaining 22 target genes were chosen for experimental analysis by our automated high-throughput dual luciferase reporter assay (Fig. 1). The computational part of HiTmIR identified 22 target genes with miR-34a-5p binding sites in the 3’UTRs of *NDUFA9, TAF4B, NRF1, POLR2J2, DNALI1, HIP1, TGM2, POLR2G, NDUFA4L2, POLR2H, SP1, POLR2E, IFT57, PPARG, HAP1, SOD2, POLR2J3, CREB3L3, POLR2F, RCOR1, DNAL4* and* UQCRFS1.*

### Analysis of the predicted miR-34a-5p target genes by HiTmIR

The respective sequences of the 22 target genes were PCR amplified using specific primers (Additional file 4: Tab. S1) and cloned into the pMIR-RNL-TK reporter vector. These reporter constructs were analyzed applying the HiTmIR-Assay. The pMIR-TCRA reporter construct, which was validated as direct target gene of miR-34a-5p in our former study, was used as positive control (Hart et al. 2018). The co-transfection of miR-34a-5p and the positive control in HEK-293T cells led to a highly significant reduction of the relative light units (RLU) to 55.7% (p < 0.001) (Additional file 2: Fig. S2A). Upon miR-34a-5p over-expression the analyzed reporter constructs exhibit RLUs ranging from 55.71% to 106.1%. The strongest significant reduction was detected for the NDUFA9_2 with 55.7%. Likewise, the RLUs of *NDUFA9, TAF4B, NRF1, POLR2J2, DNALI1, HIP1, TGM2 and POLR2G* were reduced below 85%, which was selected as threshold for a significant RLU reduction. This threshold was selected to determine positive target genes in a high confidential manner as described in our previous study (Kern et al. 2021).

**Fig. 2:**
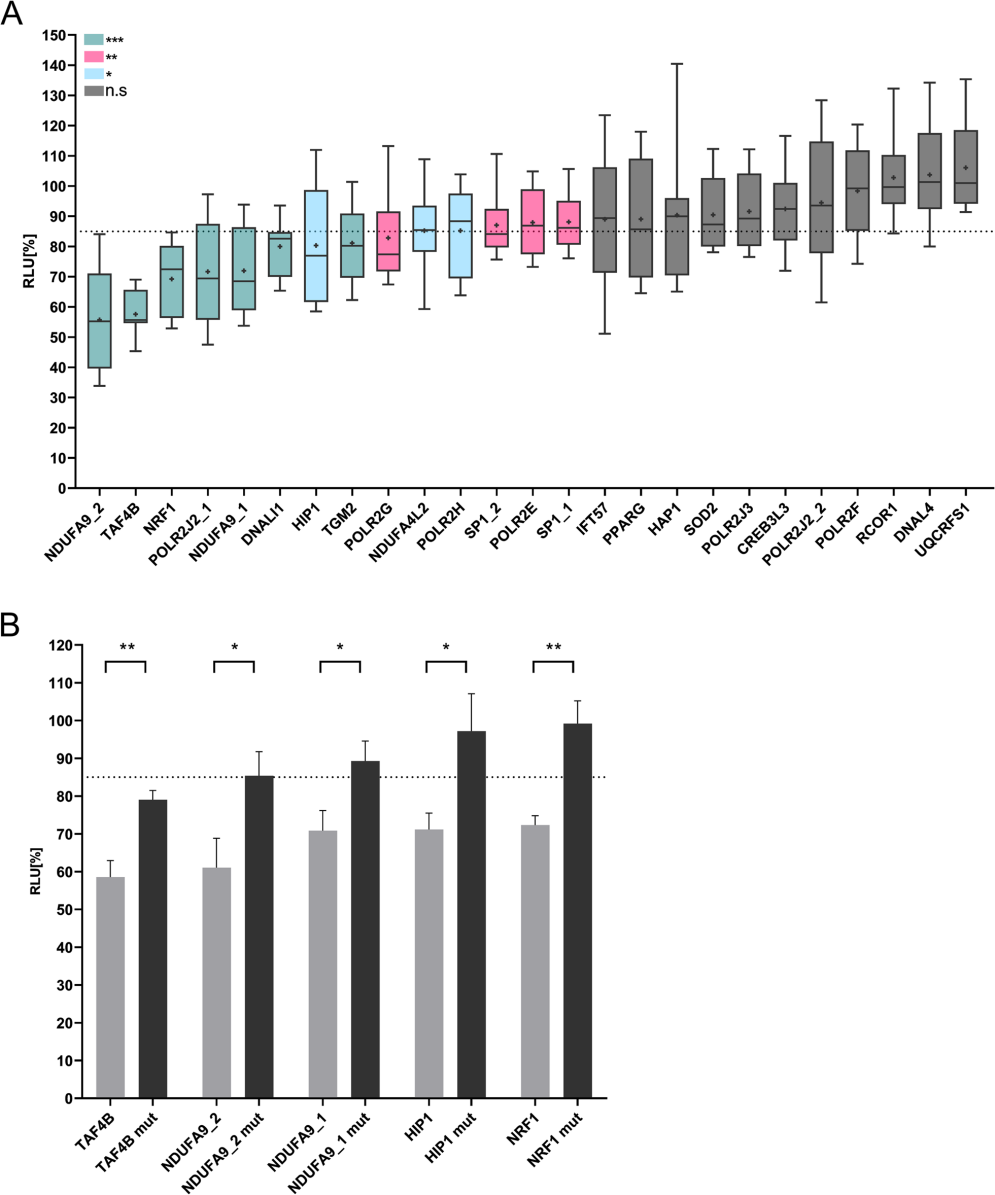
Automated dual luciferase reporter gene assays. **A** HEK 293T cells were transfected with 50 ng/well of either reporter plasmid pMIR-RNL-TK, with or without insert, and 200 ng/well of miRNA expression plasmid containing either the respective miRNA or no insert. The luciferase activities of the miR-34a transfected samples were normalized with respect to the luciferase activity measured with empty reporter plasmids. Four independent experiments were carried out in duplicates. Columns colored in turquois show a significant reduction of the luciferase activity with a p-value ≤ 0.001. Columns colored in magenta show a significant reduction of the luciferase activity with a p-value ≤ 0.01 and ≥ 0.001. Columns colored in violet show a significant reduction of the luciferase activity with a p-value ≤ 0.05. Columns colored in dark blue show a non-significant reduction of the luciferase activity with a p-value ≥ 0.05. Data are shown as mean ± SEM. **B** Dual luciferase reporter gene assays with mutated reporter plasmids. The criterion for a positive target gene was defined with a significant reduction (p-value ≤ 0.05) of the RLU of at least 15%. HEK 293 T cells were co-transfected with miR-34a-5p expression vectors and the wild type reporter plasmids of the respective target genes (light grey) or mutated reporter plasmids (mut) of the respective target genes (black) as depicted in the diagram. Four independent experiments were carried out in duplicates. Three asterisks represent a significant reduction of the luciferase activity with a p-value ≤ 0.001. Two asterisks represent a significant reduction of the luciferase activity with a p-value ≤ 0.01 and ≥ 0.001. One asterisk represents a significant reduction of the luciferase activity with a p-value ≤ 0.05. Ns indicates a non-significant reduction of the RLU

**Fig. 3:**
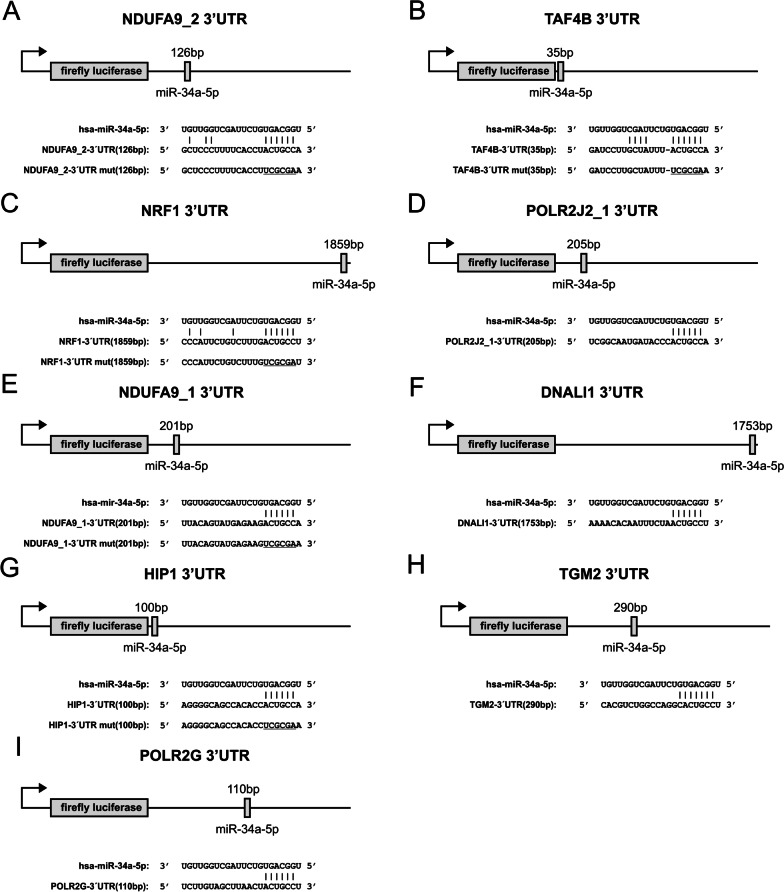
Schematic representation of reporter gene constructs. The location of the predicted miR-34a-5p binding sites in the respective 3’UTR reporter vectors, their corresponding sequences and the sequences of the respective mutated binding sites (underlined) are given. **A** NDUFA9_2-3’UTR reporter construct, **B** TAF4B-3’UTR reporter construct, **C** NRF1-3’UTR reporter construct, **D** POLR2J2_1-3’UTR reporter construct, **E** NDUFA9_1-3’UTR reporter construct, **F** DNALI1-3’UTR reporter construct, **G** HIP1-3’UTR reporter construct, **H** TGM2-3’UTR reporter construct, **I** POLR2G-3’UTR reporter construct

The reporter constructs of *NDUFA4L2, POLR2H, SP1, POLR2E, IFT57, PPARG, HAP1, SOD2, POLR2J3, CREB3L3, POLR2F, RCOR1, DNAL4* and *UQCRFS1* showed no reduction below 85% (Fig. 2A). The RLUs and p values of all initially tested reporter constructs are shown in Additional file 8: table S5. To validate the binding of miR-34a-5p to its target site we mutated the respective miR-34a-5p binding site in 3’UTRs of *TAF4B, NDUFA9_2, HIP1, NDUFA9_1* and *NRF1.* These mutated reporter constructs were tested applying our Mutagenesis HiTmIR-Assay. As described above the pMIR-TCRA reporter construct served as positive control. The co-expression of the positive control and the miR-34a expression plasmid in HEK-293 T cells led to highly significant reduction of the RLU to 52.7% (p < 0.001) (Additional file 2: Fig. S2B). All wildtype controls still displayed a significant reduction of the RLU comparable to the initial HiTmIR-Assays. All mutated reporter constructs showed a significant increase of the RLU in comparison to their respective wildtype reporter vectors (Fig. 2B) verifying the direct binding of miR-34a-5p to its binding sites in the 3’UTRs of *TAF4B, NDUFA9_2, HIP1, NDUFA9_1* and *NRF1.* The sequences and the location of the miR-34a-5p binding sites of validated target genes are shown in Fig. 3. Table 1 comprises direct miR-34a-5p target genes associated with the KEGG pathway “Huntington’s Disease” validated in the actual and former studies of our group using our HiTmIR-Assay. To verify the results of HiTmIR, we analyzed the mRNA expression of *NDUFA9, TAF4B, NRF1, POLR2J2, DNALI1, HIP1, TGM2* and *POLR2G* in two mRNA data sets. The study of Labadorf et al. investigated the mRNA expression in 20 Huntington’s Disease and 49 neurologically normal control samples (GSE64810) and the study of Lin et al. in 7 BA4 motor cortex control and 7 Huntington’s disease samples (GSE79666), both on an Illumina HiSeq 2000 platform (Labadorf et al. 2017; Lin et al. 2016). For *DNALI1* we found significantly elevated mRNA levels in the HD samples of Labadorf et al. and for *TGM2* in the HD samples of Lin et al. (Additional file 3: Fig. S3 and Additional file 9: Tab. S6). These findings are in line with the down-regulation of miR-34a-5p in HD samples of Hoss et al.. The remaining mRNAs of *NDUFA9, TAF4B, NRF1, POLR2J2, HIP1* and *POLR2G* showed no significant log2 fold changes in the HD samples pointing out on a possible regulation by miR-34a-5p on protein level by translational inhibition.

**Table 1:** Direct targets of miR-34a-5p in KEGG pathway “Huntington’s Disease”

miR-34a Target Genes in HD KEGG-Pathway	Published in	PMID
NDUFA9	This paper	n.a
TAF4B	This paper	n.a
NRF1	This paper	n.a
POLR2J2	This paper	n.a
POLR2G	This paper	n.a
DNALI1	This paper	n.a
HIP1	This paper	n.a
TGM2	This paper	n.a
COX6B2	Kern et al.	33305319
PLCB3	Kern et al.	33305319
GNAQ	Kern et al.	33305319
NDUFC2	Kern et al.	33305319
PLCB1	Kern et al.	33305319
CYCS	Kern et al.	33305319
GRIN2B	Kern et al.	33305319
UQCR11	Kern et al.	33305319
SDHC	Kern et al.	33305319
HTT	Kern et al.	33305319
ITPR2	Diener et al.	30262862
IRE1A	Krammes et al.	32531952
XBP1	Krammes et al.	32531952

### Western blot verification of miR-34a-5p target genes

**Fig. 4:**
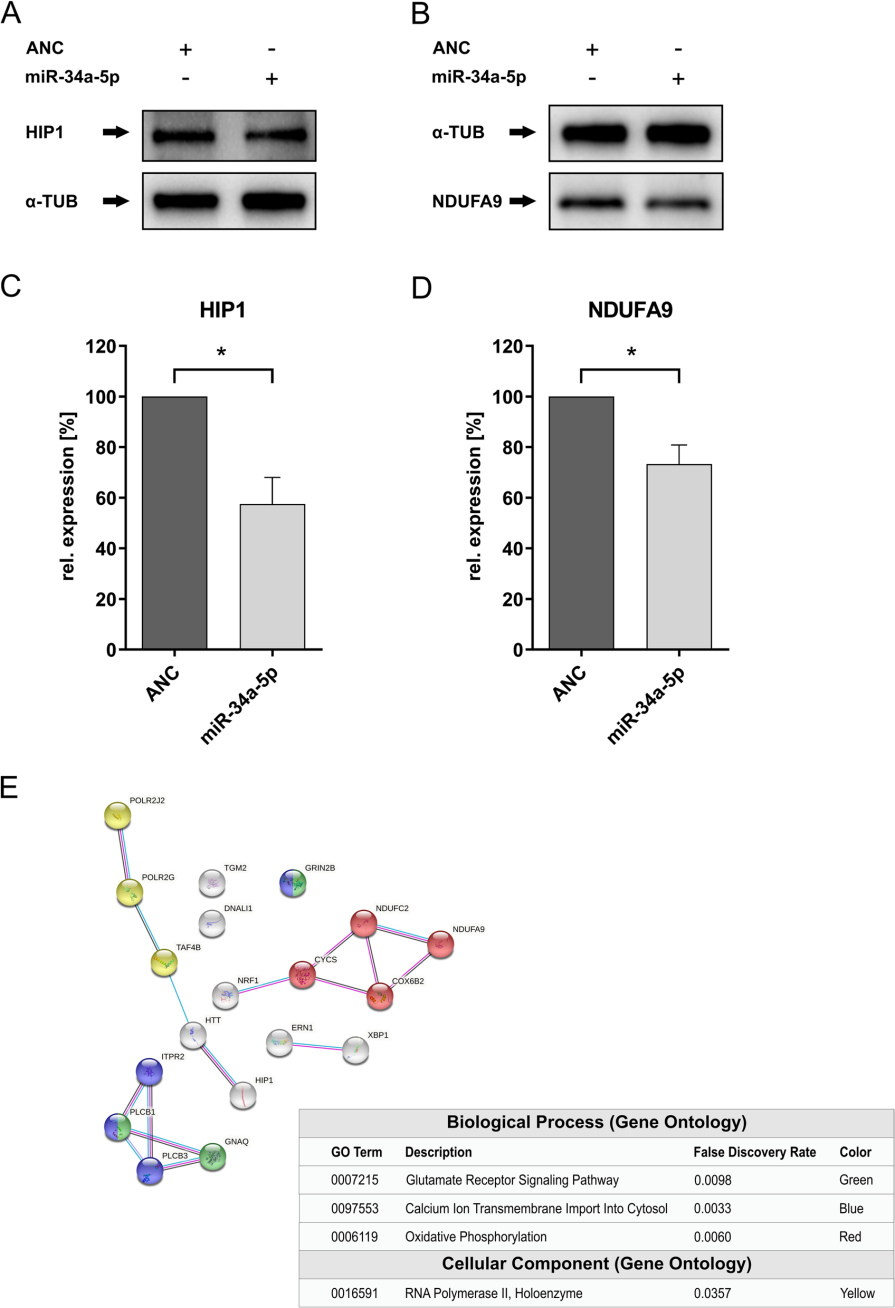
Western Blot analysis of hsa-miR-34a-5p over-expressing SH-SY5Y cells. SH-SY5Y cells were transfected either with ANC or miRNA Mimic. 48 h later, protein expression of HIP1 and NDUFA9 regulated by hsa-miR-7-5p, was detected with specific antibodies in a Western Blot analysis. The experiments were conducted in 3 independent experiments. **A** Western Blot Analysis of HIP1 protein expression regulated by hsa-miR-34a-5p over-expression. **B** Western Blot Analysis of NDUFA9 protein expression regulated by hsa-miR-34a-5p over-expression. **C** Quantitative Analysis of HIP1 protein expression regulated by hsa-miR-34a-5p over-expression. One asterisk represents a significant reduction of the luciferase activity with a p-value ≤ 0.05. **D** Quantitative Analysis of NDUFA9 protein expression regulated by hsa-miR-34a-5p over-expression. One asterisk represents a significant reduction of the luciferase activity with a p-value ≤ 0.05. **E** Protein–protein interaction networks of the 19 direct miR-34a-5p targets in Huntington’s disease using the STRING database version 11.5 (https://string-db.org/). The respective miR-34a-5p targets genes which are associated with the depicted pathways are colored as indicated in the diagram

To verify the detected miR-34a-5p target interactions on endogenous protein level, we transfected the neuronal cell line SH-SY5Y with miR-34a-5p mimic or ANC (negative control). The Western Blot analysis verified the miR-34a-5p target interactions observed by HiTmIR-Assay. We analyzed the protein levels of HIP1 and NDFUA9 using specific antibodies (Fig. 4A and B). The over-expression of miR-34a-5p in SH-SY5Y cells led to a downregulation of both analyzed proteins. Specifically, the relative expression of HIP1 was reduced to 57.5% (p = 0.01 < p ≤ 0.05) (Fig. 4C) and the relative expression of NDUFA9 reduced to 73.3% (p = 0.01 < p ≤ 0.05) (Fig. 4D).

### Protein–protein interaction network analysis of miR-34a-5p target genes linked to HD pathomechanisms

To identify distinct protein–protein interaction networks we performed a protein–protein association analysis using the STRING database with all direct miR-34a-5p target genes of Table 1, which are associated with the KEGG pathway “Huntington’s Disease” applying an interaction score of ≥ 0.4. This analysis identified four distinct interaction networks with a false discovery rate ≥ 0.05 (Fig. 4E). Three networks belong to the Gene Ontology category “Biological Process” including the subnetworks “Glutamine Receptor Signaling Pathway”, “Calcium Ion Transmembrane Import Into Cytosol” and “Oxidative Phosphorylation”. One subnetwork, “RNA Polymerase II, Holoenzyme” is categorized in the Gene Ontology category “Cellular Component” (Additional files 6, 7).

## Discussion

**Fig. 5:**
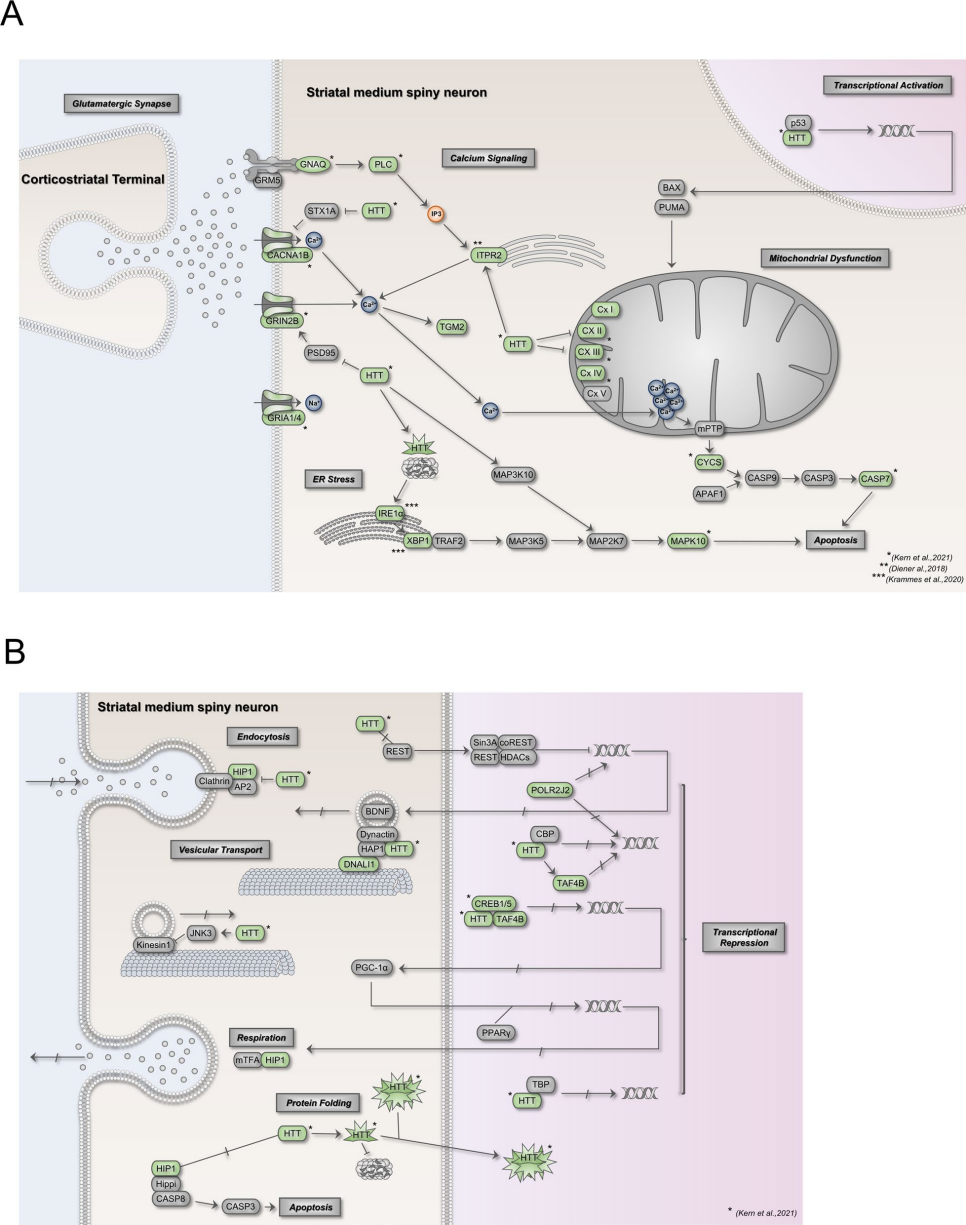
Overview of the KEGG “Huntington’s Disease Signaling Pathway” with new direct miR-34a-5p target genes. **A **Impact of miR-34a deregulation on the subcategories “glutamatergic synapse”, “calcium signaling”, “transcriptional activation”, “mitochondrial dysfunction”, “ER stress” and “apoptosis”. Direct target genes of miR-34a-5p are marked in green. All marked target genes were identified by our group. Target genes with no asterisk were identified in this study. Target genes with asterisks were identified in recent studies as indicated in the diagram (Kern et al. 2021; Diener et al. 2018; Krammes et al. 2020). **B** Impact of miR-34a deregulation on the subcategories “endocytosis”, “vesicular transport”, “transcriptional repression”, “respiration”, “protein folding” and “apoptosis”. Direct target genes of miR-34a-5p are marked in green. All marked target genes were identified by our group. Target genes with no asterisk were identified in this study. Target genes with one asterisk were identified in a recent study as indicated in the diagram (Kern et al. 2021)

Here we analyzed to role of miR-34a-5p in KEGG “Huntington’s Disease pathway” for a comprehensive understanding of miR-34a-5p associated pathomechanisms of HD. To date most studies analyzed only the impact of single miRNA target interaction on the pathogenesis of a disease. In HD there are even only a few studies dealing with the identification of miRNA target interactions. Examples include REST as target for miR-9 (Packer et al. 2008), RCOR1, RGS2, HDAC4 as targets for miR-22 (Jovicic et al. 2013), MFN2 as target for miR-214 (Bucha et al. 2015) and miR-34a-5p as modulator of the p53/SIRT1-pathway in HD (Reynolds et al. 2018). Our recent study that explored the function of miR-34a-5p on crucial Parkinson’s disease associated pathways highlighted miR-34a-5p as major modulator of complex regulation networks in neurodegenerative diseases (Kern et al. 2021). The direct miR-34a-5p targets identified in this study showed an overlap with miR-34a-5p target genes in the KEGG “Huntington’s disease pathway”, e.g., the central key factor HTT in HD. Table 1 comprises all miR-34a-5p target genes attached to the KEGG pathway “Huntington’s Disease Out of the 36 potential miR-34a-5p target genes in this HD pathway we verified 21 direct targets i.e., 58% of the predicted targets, further underlining a major role of miR-34a-5p in HD pathomechanisms. A protein–protein interaction networks analysis with the 19 verified targets of miR-34a-5p reveals miR-34a-5p as one of the key modulators of HD associated pathways like “Glutamine Receptor Signaling Pathway”, “Calcium Ion Transmembrane Import into Cytosol” and “Oxidative Phosphorylation”. To gain further insights of the affected molecular pathomechanisms, we created a pathway map of the KEGG pathway “Huntington’s Disease” and highlighted all verified miR-34a-5p target genes (Fig. 5A and B). Thereby, we found miR-34a-5p playing a role in the regulation of the “Glutamatergic Synapse”, “Calcium Signaling”, “Transcriptional Activation”, “Mitochondrial Dysfunction”, “ER Stress”, “Endocytosis”, “Vesicular Transport”, “Transcriptional Repression”, “Protein Folding”, “Respiration” and “Apoptosis”. Notably, these miR-34a-5p related pathomechanisms displayed a nearly complete overlap with the known molecular hallmarks of HD: “Aggregate Formation”, “Transcriptional Dysregulation”, “Altered Protein Homeostasis”, Mitochondrial Dysfunction”, “Altered Synaptic Plasticity”, “Axonal Transport Defect” and “Neuroglia Dysfunction”, described in the review of Jimenez-Sanchez et al. (Jimenez-Sanchez et al. 2017).

In the “Glutamatergic Synapse” pathway miR-34a-5p regulates the expression of CACNA1B (Calcium Voltage-Gated Channel Subunit Alpha1 B), GRIN2B (Glutamate Ionotropic Receptor NMDA Type Subunit 2B), GRIA1 (Glutamate ionotropic receptor AMPA type subunit 1) and GRIA4 (Glutamate ionotropic receptor AMPA type subunit 4). The down-regulation of miR-34a-5p in HD, as found in brain tissue of R6/2 mouse model, could lead to an increase of CACNA1B, GRIN2B, GRIA1 and GRIA4 protein levels in the medium spiny neurons (MSNs) enhancing their excitability. This finding is in line with Rocher et al., who found that MSNs of BACHD mice were hyperexcitable and the amplitude of AMPAR-mediated synaptic currents was higher than in WT MSNs (Rocher et al. 2016). Dysregulation of the Ca^2+^ signalosome was detected in HD models and post-mortem samples of HD patients (Hodges et al. 2006; Wu et al. 2011). We identified ITPR2 (Inositol 1,4,5-Trisphosphate Receptor Type 2), which encodes for IP_3_ receptor (IP3R) type 2, as direct target of miR-34a-5p (Diener et al. 2018). The down-regulation of miR-34a-5p in MSNs in turn results in the induction of ITPR2 protein levels being in line with an increase of the sensitivity to IP_3_. In 2013 Czeredys et al. described an elevated store-operated Ca2 + entry (SOCE) in HD models (Czeredys et al. 2017). MiR-34a-5p as key modulator of intracellular Ca^2+^ signaling contributes to the increase of SOCE in HD. Another molecular hallmark in HD pathogenesis is the dysregulation of transcription. miR-34a-5p impacts via its direct target genes POLR2J2, TAF4B, CREB1, CREB5 and HTT the transcriptional repression and via HTT in association with p53 the transcriptional activation in MSNs. McCourt and colleagues showed that CREB1 is elevated in the adipose tissue of HD patients (McCourt et al. 2015). The down-regulation of miR-34a-5p could contribute to the elevated CREB1 levels in HD patients. Mitochondrial dysfunction is one of the major hallmarks of HD and almost all neurodegenerative diseases. miR-34a-5p targets key components of the respiratory chain complexes (RCC) I to IV. In detail, miR-34a-5p regulates the expression of NDUFA9 and NDUFC2 (RCCI), SDHC (RCCII), UQCR11 (RCCIII) as well as COX6B2 (RCCIV). As direct targets of down-regulated miR-34a-5p all these target genes will be induced resulting in an increase of ATP depletion and reactive oxygen species (ROS). In the apoptosis pathway miR-34a-5p regulates the expression of CYCS and CASP7. The major caspase activation pathway is initiated by CYCS release into the cytosol (Jiang and Wang 2004). In addition, the polyglutamine expansions of mHTT disturb the ER formation leading to CASP7 activation (Ueda et al. 2014). ER Stress caused by accumulation of misfolded proteins is discussed as one of the leading pathomechanisms for HD (Kalathur et al. 2015). As counterpart of ER Stress, the unfolded protein response (UPR) is activated. MiR-34a-5p is shown to modulate also the IRE1A branch of the UPR leading to enhanced activation of apoptosis by downregulation of miR-34a-5p via MAPK10. One of the hallmarks of HD is the dysregulation of the axonal transport. miR-34a-5p regulates the expression of *HIP1*, *DNALI1* and *HTT* posttranscriptionally with impact on the pathways “Endocytosis” and “Vesicular Transport”. Low levels of miR-34a-5p in MSNs are accompanied by higher levels of HIP1, which in turn could led to an enhanced NMDA-induced excitotoxicity (Metzler et al. 2007). Forming a complex with HIPPI, HIP1 regulates apoptosis by downstream activation of caspases resulting in an induction of apoptosis (Hackam et al. 2000; Choi et al. 2006). Additionally, mHTT itself is a direct target of miR-34a-5p (Kern et al. 2021). This could contribute to higher levels of mHTT protein in MSNs with impact on Glutamatergic Synapse”, “Calcium Signaling”, “Transcriptional Activation”, “Mitochondrial Dysfunction”, “ER Stress”, “Endocytosis”, “Vesicular Transport”, “Transcriptional Repression”, “Protein Folding” and “Apoptosis”.

## Conclusions

Our comprehensive analysis of the impact of miR-34a-5p downregulation on KEGG “Huntington’s disease” pathway identifies miR-34a-5p as one of the key modulators of HD associated pathomechanisms. We demonstrate that miR-34a-5p has direct target genes in a variety of pathways like “Glutamatergic Synapse”, “Calcium Signaling”, “Transcriptional Activation”, “Mitochondrial Dysfunction”, “ER Stress”, “Endocytosis”, “Vesicular Transport”, “Transcriptional Repression”, “Protein Folding”, “Respiration” and “Apoptosis”. Taken together, the downregulation of miR-34a-5p in MSNs of R6/2 mice and human HD brain samples might contribute to an elevated intracellular Ca^2+^ level, increased UPR and ROS levels resulting in a pronounced activation of apoptosis. Our results lay the ground for future therapeutic approaches to reconstitute the basal level of miR-34a-5p in MSNs, while the specific administration of dedicated amounts of miR-34a-5p mimics to MSNs is of crucial importance.

## Supplementary Information

**Additional file 1: Figure S1.** Re-analysis of next generation small RNA sequencing data of Hoss et al. for miR-34a-5p expression. Two asterisks represent a significant reduction of miR-34a-5p expression with a p-value ≤ 0.01 and ≥ 0.001.

**Additional file 2: Figure S2.** (A): Results of controls of automated dual luciferase assay. Empty expression plasmid (pSG5) and reporter plasmid (pMIR) as well as positive control (TCRA) and miRNA expression plasmid for hsa-miR-34a-5p (miR-34a) were transfected in 293 T cells in the indicated combinations. The experiments were carried out in four independent experiments in technical duplicates. Three asterisks represent a significant reduction of the luciferase activity with a p-value ≤ 0.001. Ns indicates a non-significant reduction of the RLU. (B): Results of controls of automated dual luciferase assay with the mutated reporter constructs. Empty expression plasmid (pSG5) and reporter plasmid (pMIR) as well as positive control (TCRA) and miRNA expression plasmid for hsa-miR-34a-5p (miR-34a) were transfected in 293 T cells in the indicated combinations. The experiments were carried out in four independent experiments in technical duplicates. Three asterisks represent a significant reduction of the luciferase activity with a p-value ≤ 0.001. Ns indicates a non-significant reduction of the RLU.

**Additional file 3: Figure S3. **mRNA expression of *NDUFA9, TAF4B, NRF1, POLR2J2, DNALI1, HIP1, TGM2* and *POLR2G* in HD brain samples of Labadorf et al. and Lin et al.. Labadorf et al. investigated the mRNA expression in 20 Huntington's Disease and 49 neurologically normal control samples (GSE64810) and Lin et al. in 7 BA4 motor cortex control and 7 Huntington's disease samples (GSE79666), both on an Illumina HiSeq 2000 platform.

**Additional file 4: Supplemental Table 1.** Cloning primers for cloning of target gene 3’UTRs

**Additional file 5: Supplemental Table 2.** Mutagenesis primers for mutation of miR-34a-5p target sites.

**Additional file 6: Supplemental Table 3:** GeneTrail3 pathway analysis of predicted miR-34a-5p target genes in KEGG pathways

**Additional file 7: Supplemental Table 4:** Predicted miR-34a-5p target genes in KEGG pathway “Huntington’s Disease”.

**Additional file 8: Supplemental Table 5:** RLUs and p values of all initially tested 3’UTR-sequences.

**Additional file 9: Supplemental Table 6:** Raw data of the re-analysis of next generation mRNA sequencing data of Labadorf et al. and Lin et al.

## Data Availability

All data generated or analyzed during this study are included in this published article and its additional information files.

## Abbreviations

HD: Huntington’s disease
miR: MicroRNA
KEGG: Kyoto Encyclopedia of Genes and Genomes
NDUFA9: NADH:ubiquinone oxidoreductase subunit A9
TAF4B: TATA-box binding protein associated factor 4b
NRF1: Nuclear respiratory factor 1
POLR2J2: RNA polymerase II subunit J2
DNALI1: Dynein axonemal light intermediate chain 1
HIP1: Huntingtin interacting protein 1
TGM2: Transglutaminase 2
POLR2G: RNA polymerase II subunit G
STRING: Search Tool for the Retrieval of Interacting Genes/Proteins
HTT: Huntingtin
mHTT: Mutant Huntingtin
HiTmIR: High-throughput miRNA interaction reporter assay
DSMZ: German collection of microorganisms and cell cultures
DMEM: Dulbecco’s Modified Eagle Medium
FCS: Fetal calf serum
3’UTR: 3’ Untranslated region
ANC: All star negative control
MFN2: Mitofusin 2
CACNA1B: Calcium voltage-gated channel subunit Alpha1 B
GRIN2B: Glutamate ionotropic receptor NMDA type subunit 2B
GRIA1: Glutamate ionotropic receptor AMPA type subunit 1
GRIA4: Glutamate ionotropic receptor AMPA type subunit 4
MSN: Medium spiny neuron
ITPR2: Inositol 1,4,5-Trisphosphate Receptor Type 2
SOCE: Store-operated Ca2 + entry
POLR2J2: RNA polymerase II subunit J2
CREB1: CAMP responsive element binding protein 1
CREB5: CAMP responsive element binding protein 5
ROS: Reactive oxygen species
CYCS: Cytochrome c, somatic
CASP7: Caspase 7
NMDA: N-methyl-d-aspartate
UPR: Unfolded protein response
