# Supplementary figures and images for: miR-34a-5p as molecular hub of pathomechanisms in Huntington’s disease
Source: Mol Med. 2023 Apr 3;29:43. doi: 10.1186/s10020-023-00640-7 (PMC10295337; doi:10.1186/s10020-023-00640-7)

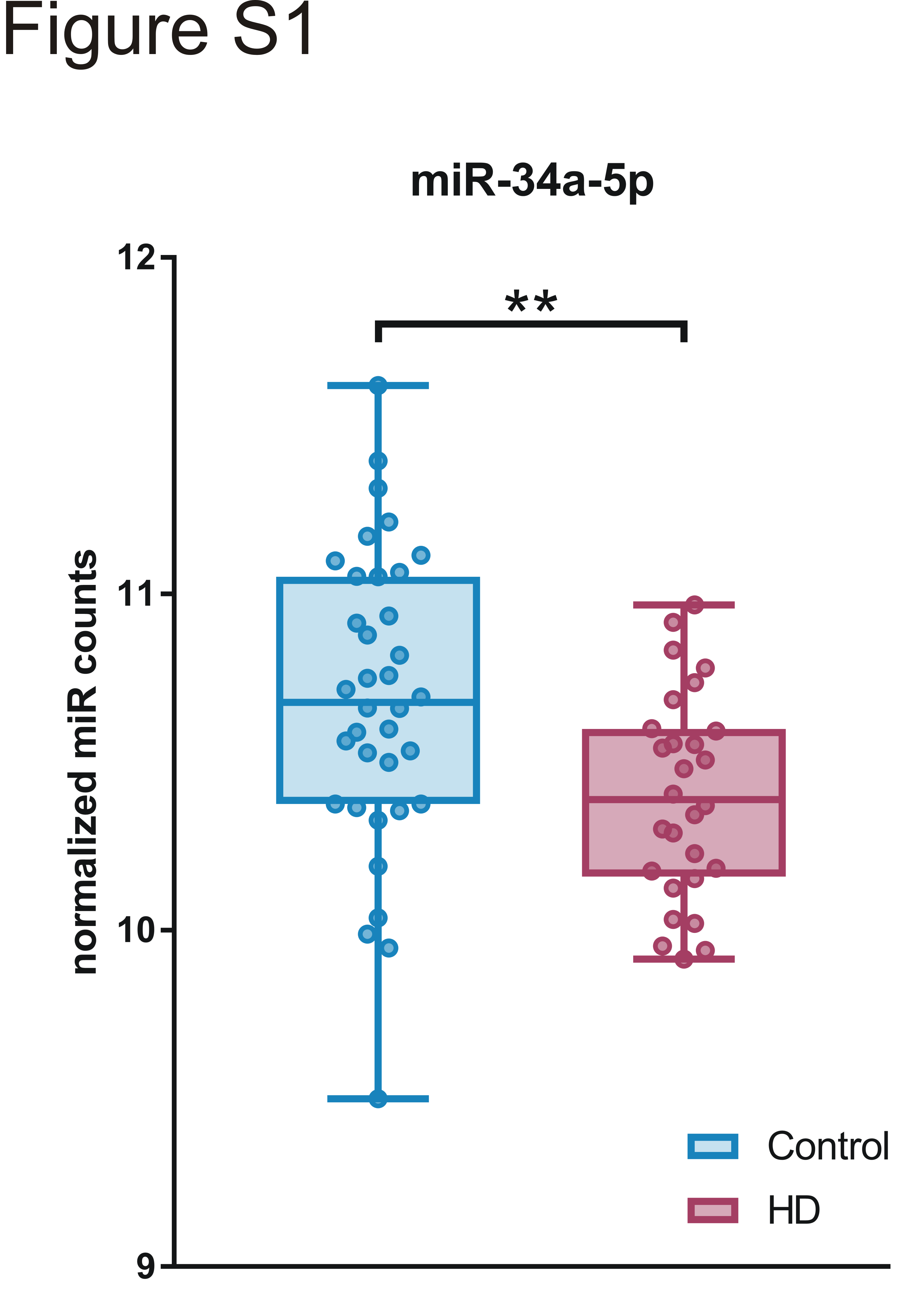

Supplement: Supplementary file 1 — Additional file 1: Figure S1. Re-analysis of next generation small RNA sequencing data of Hoss et al. for miR-34a-5p expression. Two asterisks represent a significant reduction of miR-34a-5p expression with a p-value ≤ 0.01 and ≥ 0.001. [file 10020_2023_640_MOESM1_ESM.tif]

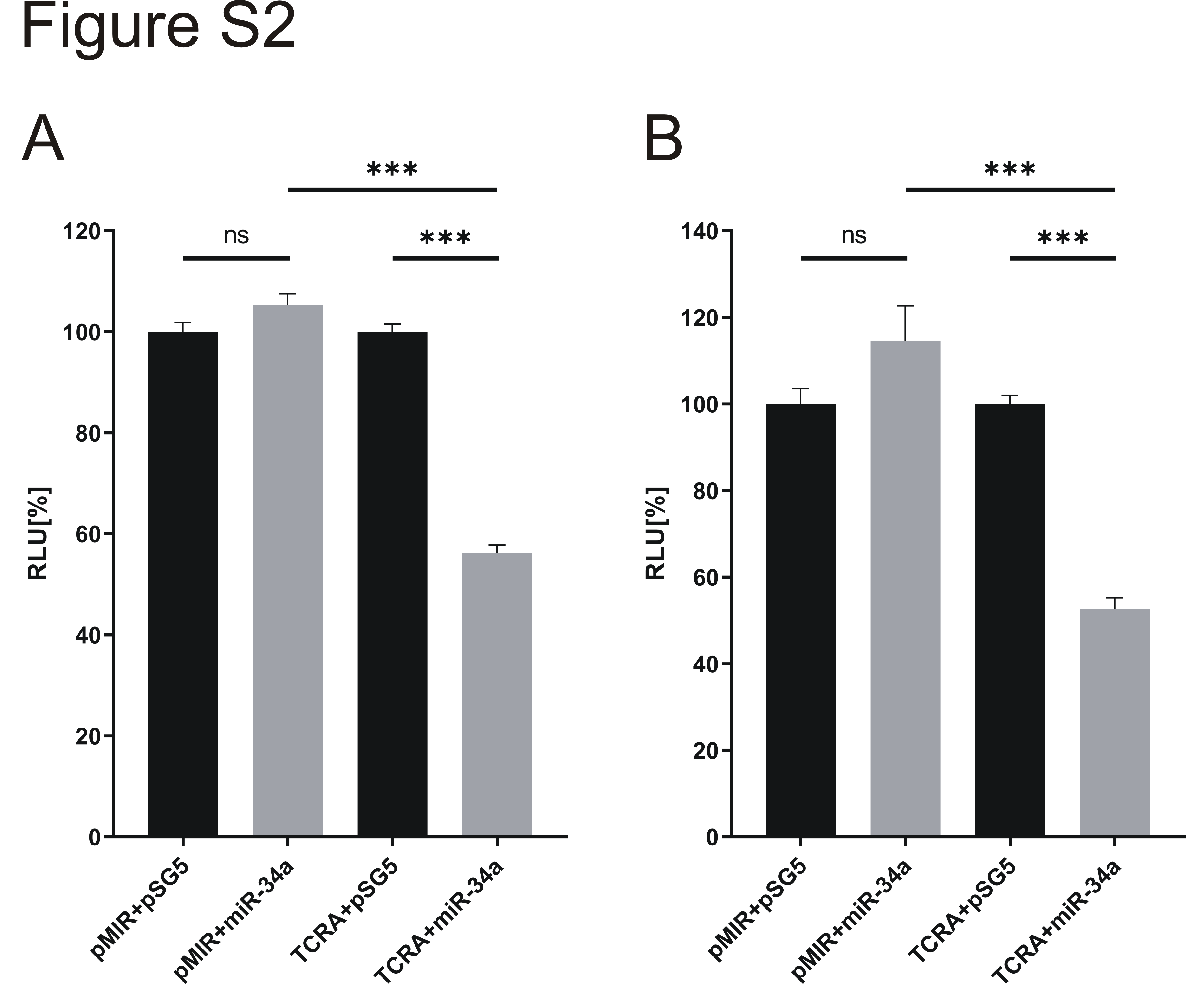

Supplement: Supplementary file 2 — Additional file 2: Figure S2. (A): Results of controls of automated dual luciferase assay. Empty expression plasmid (pSG5) and reporter plasmid (pMIR) as well as positive control (TCRA) and miRNA expression plasmid for hsa-miR-34a-5p (miR-34a) were transfected in 293 T cells in the indicated combinations. The experiments were carried out in four independent experiments in technical duplicates. Three asterisks represent a significant reduction of the luciferase activity with a p-value ≤ 0.001. Ns indicates a non-significant reduction of the RLU. (B): Results of controls of automated dual luciferase assay with the mutated reporter constructs. Empty expression plasmid (pSG5) and reporter plasmid (pMIR) as well as positive control (TCRA) and miRNA expression plasmid for hsa-miR-34a-5p (miR-34a) were transfected in 293 T cells in the indicated combinations. The experiments were carried out in four independent experiments in technical duplicates. Three asterisks represent a significant reduction of the luciferase activity with a p-value ≤ 0.001. Ns indicates a non-significant reduction of the RLU. [file 10020_2023_640_MOESM2_ESM.tif]

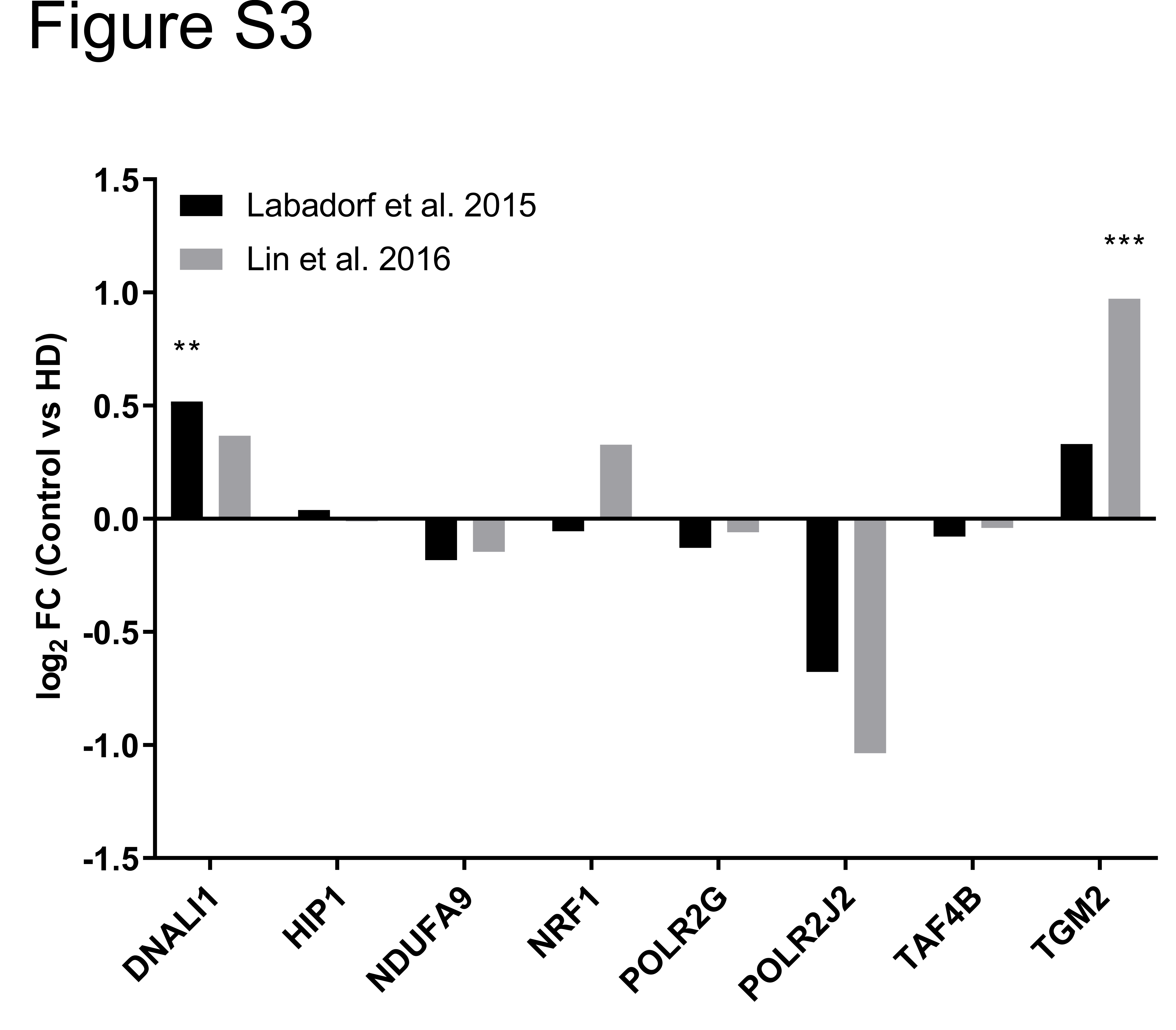

Supplement: Supplementary file 3 — Additional file 3: Figure S3. mRNA expression of NDUFA9, TAF4B, NRF1, POLR2J2, DNALI1, HIP1, TGM2 and POLR2G in HD brain samples of Labadorf et al. and Lin et al.. Labadorf et al. investigated the mRNA expression in 20 Huntington's Disease and 49 neurologically normal control samples (GSE64810) and Lin et al. in 7 BA4 motor cortex control and 7 Huntington's disease samples (GSE79666), both on an Illumina HiSeq 2000 platform. [file 10020_2023_640_MOESM3_ESM.tif]
